# Effect of clinical and histologic features on time to malignancy in 224 cases of oral leukoplakia treated by surgery

**DOI:** 10.1007/s00784-022-04486-x

**Published:** 2022-04-26

**Authors:** Jose Bagan, Miguel Martorell, Jose L. Cebrián, Andrea Rubert, Leticia Bagán, Carlos Mezquida, David Hervás

**Affiliations:** 1 Deparment of Stomatology, Valencia University, C/ Gasco Oliag 1, 46010 Valencia, Spain; 2 University General Hospital, Valencia, Spain; 3 Valencia University, Valencia, Spain; 4 Hospital Universitario la Paz, Madrid, Spain; 5 Universidad Europea de Valencia, Valencia, Spain; 6 Department of Applied Statistics, Operational Research and Quality, Universitat Politècnica de València, Valencia, Spain

**Keywords:** Oral leukoplakia, Malignancy, Evolution

## Abstract

**Objectives:**

Our main purpose and research question were to analyze and quantify whether there were significant differences in the time to develop cancer among patients with oral leukoplakia (OL), comparing the more susceptible cases to those with the least susceptibility to malignancy.

**Materials and methods:**

We followed 224 cases of OL after surgical or CO_2_ laser treatment for a mean time of 6.4 years. A Bayesian mixture cure model based on the Weibull distribution was used to model the relationship between our variables and cancer risk. In this model type, the population is considered a mixture of individuals who are susceptible or non-susceptible to developing cancer. The statistical model estimates the probability of cure (incidence model) and then infers the time to malignancy. The model was adjusted using the R-package INLA using default priors.

**Results:**

Histology type (moderate or severe dysplasia) and tongue location showed hazard ratios (HR) of 3.19 (95% CI [1.05–8.59]) and 4.78 (95% CI [1.6–16.61]), respectively. Both variables increased the risk of malignant transformation, thus identifying a susceptible subpopulation with reduced time required to develop cancer, as with non-homogeneous leukoplakias. The median time for cancer development was 4 years and 5 months, with a minimum of 9 months after the diagnosis of OL and a maximum of 15 years and 2 months.

**Conclusions:**

Susceptible patients with non-homogeneous leukoplakia, dysplasia, or leukoplakia in the tongue develop cancer earlier than those with homogeneous OL and those without dysplasia.

**Clinical relevance:**

The novel contribution of this research is that, until now, the time it took for oral leukoplakias to develop cancer based on whether they were homogeneous or non-homogeneous, and if they have or not epithelial dysplasia, had not been comparatively described and quantified. As a final result, the time to malignant transformation in non-homogeneous and dysplastic leukoplakias is significantly shorter.

## Introduction

Oral leukoplakia (OL) is the most common and well-known potentially malignant oral disorder. International consensus proposals have been made to define it. One of the best and most cited definitions in the international literature was that established in 2007 by Warnakulasuriya, Johnson, and van der Waal, as part of an international working group that met in London in 2005, coordinated by the WHO Collaborating Centre for Oral Cancer and Precancer in the UK. They defined OL as white plaques of questionable risk, having excluded other known diseases or disorders that carry no increased risk for cancer. Furthermore, leukoplakia is a clinical term, and the lesion has no specific histology [1].

In March 2020, the WHO Collaborating Centre for Oral Cancer in the UK convened a workshop to discuss the oral potentially malignant disorders (OPMDs). Regarding oral leukoplakia, the group found no reasons to change the definition published in 2007 [2].

Regarding the malignant transformation (MT) of OL, Holmstrup et al. [3] reported that in surgically treated OL, 12% developed cancer after a follow-up period of 7.5 years; however, when the lesions were not surgically treated, 4% developed malignancy after a mean observation period of 6.6 years.

Warnakulasuriya et al. [4], in a systematic review of observational studies, reported that the estimated overall (mean) MT rate for OL was 3.5%, with a wide range between 0.13 and 34.0% (4). According to Aguirre-Urizar et al. [5], the pooled proportion of malignant transformation MT was 9.8% (95% CI: 7.9–11.7,5).

Most authors agree that for non-homogeneous leukoplakia [4, 6–10], the size of the lesions [9, 11, 12], the location of the lesions in the most common risk area for cancer on the tongue [7, 11, 13], and the presence of epithelial dysplasia [9, 11, 14, 15] are associated with an increased risk of developing oral cancer during follow-up.

It is well known that homogeneous leukoplakia, small lesions, and non-dysplastic leukoplakias may progress to cancer during follow-up, but this is less frequent. As Villa and Woo [16] stated, any leukoplakia of whatever clinical and histopathological appearance may become malignant. However, the predictive influence of the clinical and histological findings in every case, regarding the possible MT of OL, varies among reports. This is why it is important, as Aguirre-Urizar et al. [5] pointed out, to continue performing well-designed prospective clinicopathological studies of OL, using a uniform definition of OL to reduce the risk of bias when evaluating factors associated with MT.

Many authors have analyzed the possible clinical and histological variables that predispose to MT as we described above; however, to the best of our knowledge, the difference in time to development of cancer between cases with factors that predict a high risk, such as non-homogeneous OL and epithelial dysplasia, and cases with a lower risk, such as homogeneous leukoplakia and no dysplasia, has not been described in a large number of OL cases. This was the main and original objective of our present study.

## Materials and methods

For the study, we selected a group of 224 patients with OL who visited and were treated at the Stomatology and Maxillofacial Surgery Service of the Hospital General Universitario de Valencia between 1994 and 2018, all of whom met the diagnostic criteria established by Warnakulasuriya et al. (2007) [2] to establish the definitive diagnosis of OL. The study was approved by the Ethics Committee of the Universitat de Valencia (procedure number H1456655015143).

Cases with homogeneous and non-homogeneous leukoplakia were included, but proliferative verrucous leukoplakia was excluded. Patients whose histopathologic results at the time of initial diagnosis and after the first biopsy were carcinoma in situ and/or oral squamous cell carcinoma were also excluded. We also excluded cases that did not consent to be treated to eliminate the lesion (i.e., those who only accepted follow-up of the lesions without treatment). Cases that did not have at least 1 year of follow-up after the initial diagnosis were also excluded.

In all patients, the treatment was surgery with removal of the lesion or CO2 laser treatment (vaporization at 15 W); therefore, no patient was left without treatment to remove the lesion. First and before any treatment, we always took not one but if necessary several biopsies of the different areas of the leukoplakia before laser vaporization. In addition, in high-grade dysplasia, our tendency is to perform surgical excisions with histological examination of the entire lesion and vaporization is usually indicated when there is no or low-grade dysplasia.

Patients were followed up for a mean of 6.4 years (4.9 years standard deviation), with a minimum of 1 year and a maximum of 20.8 years of follow-up. Each patient was reviewed at intervals between 3 and 6 months depending on their risk factors.

The presence of lesions was recorded at the first visit, indicating the clinical type, location, and size of the lesions. Once the initial biopsy was taken, we indicated whether there was dysplasia and the degree of dysplasia (mild, moderate, or severe). We also grouped cases with no dysplasia and mild dysplasia as low grade, while those with moderate or severe dysplasia were grouped as high grade, according to Odell et al. [17].

If an oral squamous cell carcinoma developed, we indicated the time elapsed from the initial diagnosis of leukoplakia to the diagnosis of carcinoma.

### Statistical analysis

Data were summarized using the mean (standard deviation) and median (1st, 3rd quartile) in the case of numerical variables and with relative and absolute frequencies in the case of categorical variables. To assess the associations between the different study variables and the risk of developing cancer, a survival analysis was performed. Since many leukoplasia patients will never develop cancer, a Bayesian mixture cure model based on the Weibull distribution was used to model the relationship between our variables and the risk of cancer. In this type of model, the population is considered a mixture of susceptible and non-susceptible individuals. The model estimates the probability of a cure (incidence model) and then makes an inference on the time to event in the susceptible individuals (latency model). The model was adjusted using the R-package INLA (version 21.11.22) with default priors. Predictors considered in the model were clinical type, histology, size, location in gingiva, location in tongue, treatment, and smoking status. For all parameters in the model, 95% credibility intervals were estimated [18].

## Results

In our sample of 224 patients with OL, whose clinical and histopathological data are shown in Table 1, 26 cases (11.6%) progressed to cancer after the date of the first diagnosis and after follow-up. In those who developed cancer, the mean time to cancer development was 6 years (4.5 years). The median time was 4 years and 5 months, with a minimum of 9 months after the diagnosis of OL and a maximum of 15 years and 2 months. The evolution of our 224 patients was as follows: 82 (36.6%) were cured after surgical treatment and 116 (51.7%) had recurrences.

**Table 1:** Clinical and histological characteristics of 224 oral leukoplakias, comparing cases with or without malignant transformation

Variable	No cancer development (*N* = 198) Mean (SD)/*n* (%) Median (1st, 3rd Q.)	Cancer development (*N* = 26) Mean (SD)/*n* (%) Median (1st, 3rd Q.)
Follow-up (years)	5.73 (4.41)	11.9 (5.54)
	4.25 (2.27, 7.86)	12.02 (8.56, 15.52)
Age (years)	55.63 (12.98)	59.15 (14.18)
	56 (47, 64)	61.5 (51.5, 69.5)
Size (mm)	19.22 (15.49)	20.27 (13.25)
	15 (10, 25)	20 (10, 20)
Gender		
Male	93 (46.97%)	11 (42.31%)
Female	105 (53.03%)	15 (57.69%)
Tobacco	102 (51.52%)	8 (30.77%)
Alcohol	31 (15.66%)	4 (15.38%)
Location		
Tongue	57 (28.79%)	21 (80.77%)
Gingiva	83 (41.92%)	2 (7.69%)
Palate	23 (11.62%)	1 (3.85%)
Floor mouth	25 (12.63%)	2 (7.69%)
Buccal mucosa	59 (29.8%)	5 (19.23%)
Clinical type		
Homogeneous	168 (84.85%)	13 (50.0%)
Erythroleukoplakia	4 (2.02%)	4 (15.38%)
Nodular	8 (4.04%)	5 (19.23%)
Verrucous	18 (9.09%)	4 (15.38%)
Histological findings		
Without dysplasia	132 (66.67%)	11 (42.31%)
Mild dysplasia	50 (25.25%)	7 (26.92%)
Moderate dysplasia	15 (7.58%)	7 (26.92%)
Severe dysplasia	1 (0.51%)	1 (3.85%)
Surgically treated	153 (77.27%)	19 (73.08%)
Laser treated	45 (22.73%)	7 (26.92%)

When comparing both groups within our sample, sex (53.03% female in non-cancer vs. 57.69% female in cancer) and type of treatment (22.73% laser vs. 26.92% non-laser) were balanced between those who developed cancer and those who did not. However, the group with cancer development had a smaller proportion of smokers (51.52% vs. 30.77%) and a smaller proportion of lesions located in the gingiva (41.92% vs. 7.69%). On the other hand, the non-cancer group had a smaller proportion of lesions located in the tongue (28.79% vs. 80.77%). The distribution of clinical types was also unbalanced, with the group developing cancer showing a higher proportion of non-homogeneous types (15.15% vs. 50%). Finally, the group developing cancer had a higher proportion of moderate and severe dysplasia at first visit when compared to the non-cancer group (7.58% vs. 26.92% and 0.51% vs. 3.85%, respectively).

**Fig. 1:**
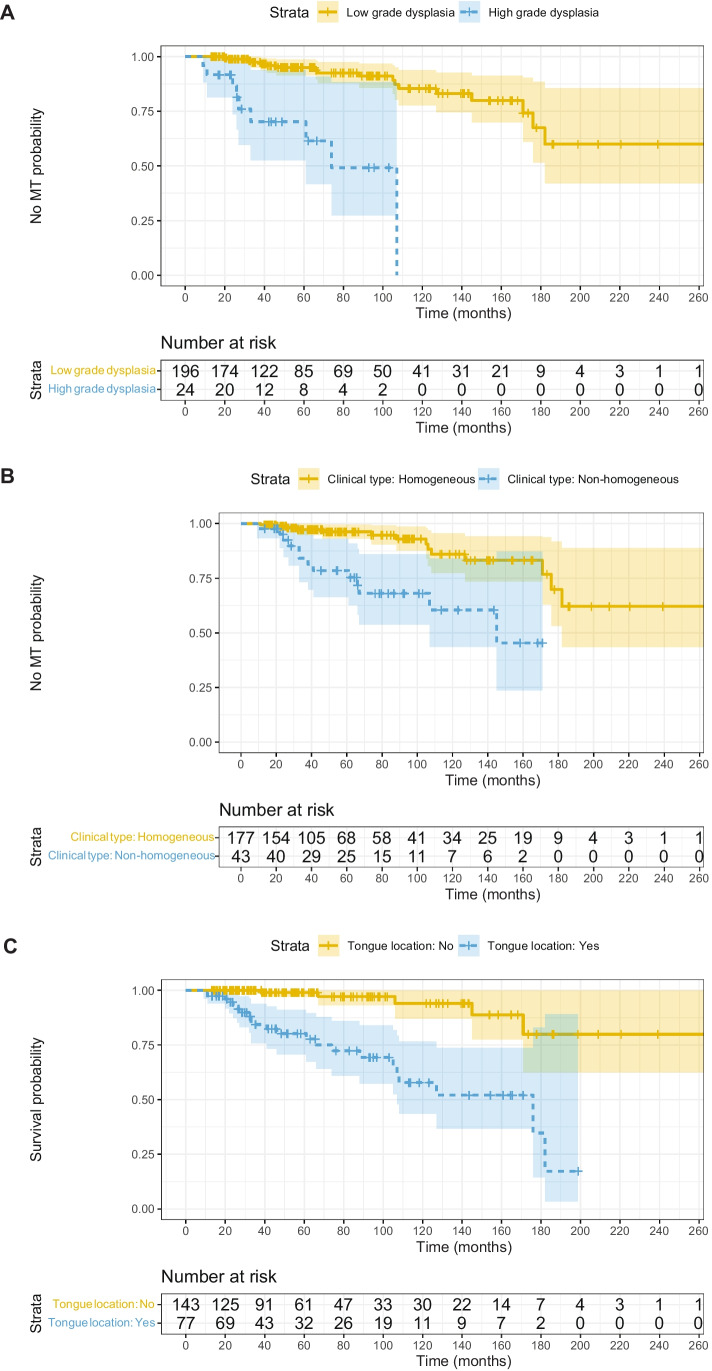
Survival curves for the three variables (**A** dysplasia, **B** clinical type, and **C** tongue location of oral leukoplakia)

To perform the survival analysis based on the cure model, we considered the variables clinical type, histology, size, location in tongue, location in gingiva, treatment, and smoking status. The results of the model are presented in Table 2. The estimate for the proportion of non-susceptible patients was 0.27 (95% CI [0.16–0.48]). Histology type (moderate or severe dysplasia) and location in the tongue showed hazard ratios (HRs) of 3.19 (95% CI [1.05–8.59]) and 4.78 (95% CI [1.6–16.61]), respectively. Since the 95% CI for the coefficients of both variables excluded 1, it can be concluded that both variables increase the risk of MT, thus reducing the time required to develop cancer in the susceptible subpopulation. There was also a non-significant amount of evidence regarding the increase in risk of MT for the non-homogeneous clinical subtype, with a HR estimate of 2.37 (95% CI [0.94–6.35]), although the credible interval did not exclude zero with a probability of 95% but 94%. Results for the variables gingiva location, treatment, and tobacco provided zero or mild evidence of any effect regarding the risk of MT. For aid in the interpretation of our results, we produced survival curves for the three variables (Fig. 1).

**Table 2:** Survival analysis of oral leukoplakias based on the cure model

Variables	Estimate	SD	HR	2.5 Q	97.5 Q
(Intercept)	− 9.09	1.33	-	− 11.78	− 6.66
Clinical type (non-homogeneous)	0.86	0.48	2.37	0.94	6.35
Histology (high-grade dysplasia)	1.16	0.53	3.19	1.05	8.59
Size	0.015	0.01	1.02	0.98	1.04
Location tongue	1.57	0.60	4.78	1.60	16.61
Location gingiva	− 1.21	0.81	0.30	0.05	1.26
Treatment (laser)	− 0.47	0.52	0.63	0.23	1.73
Tobacco	− 0.01	0.48	0.99	0.38	2.45
Cure fraction	0.27	0.09	-	0.16	0.48

Estimates for the effects of the different variables are presented along with their standard errors, Hazard ratios and their corresponding 95% confidence intervals. The models also provide an estimate for the cure fraction, which stands for the estimated proportion of the patients that will never experience MT

As a sensitivity analysis, a secondary model was fitted including only the variables histology, location in the tongue, and clinical type as predictors. This model yielded similar estimates to those of our primary model. Its results can be reviewed in Table 3. Table 4 shows the characteristics of oral squamous cell carcinomas developed after follow-up of our 224 leukoplakias.

**Table 3:** Survival analysis of oral leukoplakias based on our primary model

Variables	Estimate	SD	HR	2.5 Q	97.5 Q
(Intercept)	− 9.09	1.12	-	− 11.78	− 6.66
Clinical type (non-homogeneous)	0.83	0.50	2.29	0.89	6.44
Histology (high-grade dysplasia)	1.23	0.54	3.42	1.12	3.48
Location tongue	1.98	0.53	7.24	2.75	22.2
Cure fraction	0.17	0.12	-	0.02	0.47

Estimates for the effects of the different variables are presented along with their standard errors, hazard ratios, and their corresponding 95% confidence intervals. The models also provide an estimate for the cure fraction, which stands for the estimated proportion of the patients that will never experience MT

**Table 4:** Characteristics of oral squamous cell carcinomas developed from oral leukoplakias

Case	Age	Gender	Location of OSCC	TNM	Recurrence after treatment
1	66	Female	Buccal mucosa	T2N3bM0	Yes
2	74	Female	Gingiva	T1N0M0	Yes
3	60	Male	Tongue	T2N0M0	Yes
4	43	Male	Tongue	T2N0M0	Yes
5	80	Male	Gingiva	T2N3bM0	Yes
6	78	Female	Gingiva	T3N0M0	Yes
7	73	Male	Tongue	T1N0M0	Yes
8	78	Female	Gingiva	T1N0M0	Yes
9	59	Male	Gingiva	T1N0M0	Yes
10	67	Male	Floor of mouth	T1M0N0	No
11	27	Male	Tongue	TisN0M0	Yes
12	85	Female	Tongue	T2N2aM0	Yes
13	64	Female	Tongue	T1N0M0	Yes
14	62	Male	Tongue	T1N0M0	No
15	74	Female	Tongue	T1N0M0	Yes
16	79	Female	Tongue	T2N0M0	Yes
17	45	Male	Buccal mucosa	T1N0M0	No
18	66	Female	Tongue	T1N0M0	No
19	63	Male	Lower lip	TisN0M0	No
20	48	Female	Tongue	T1N0M0	No
21	53	Male	Tongue	T1N0M0	No
22	72	Female	Tongue	T2N0M0	Yes
23	76	Female	Tongue	T2N1M0	No
24	59	Female	Gingiva	T4aN0M0	Yes
25	89	Female	Tongue	T1N0M0	No
26	72	Male	Tongue	T2N0M0	Yes

*OSCC*, oral squamous cell carcinoma; *TNM*, *TNM Classification of Malignant Tumours*

## Discussion

OL, as a frequent and potentially malignant lesion, has been widely described in the medical literature. However, OL has a rate of MT that varies widely in different studies, ranging from 1.1% [19] up to 40.8% [7].

In our study of 224 patients, whom we followed for an average of 6 years, we found 26 (11.6%) with MT, which is very similar to that described by Holmstrup et al. [3], who reported 12%. These are higher figures than the 3.5% presented in the meta-analysis by Warnakulasuriya et al. [4]. Aguirre-Urizar et al. [5] found an overall proportion of MT of 9.8% (95% CI: 7.9–11.7). Tovaru et al. [20] reported 7.5% malignant transformation in their 120 leukoplakias, being more frequent in women and in those with dysplasia. Schepman et al. [21] described a 2.9% annual malignant transformation rate in their 166 patients and Warnakulasuriya et al. [22] informed that 2.6% of OPMDs cases transformed to invasive cancer. Napier et al. [23] found that size was the most important clinical factor in determining future OSCC risk in OPMDs, especially when multiple consecutive anatomical sites were affected.

This wide range reflects the diverse criteria used in different studies. The use of uniform inclusion and exclusion criteria when selecting patients for retrospective and prospective studies on OL should lead to more consistent findings.

It is important for clinicians to know, a priori, which clinical and histological data predispose to an increased risk of oral cancer. In this regard, many authors have described and analyzed these factors. Holmstrup et al. [3] reported that the risk of malignant development of non-homogeneous leukoplakia was higher (OR = 7.0) than that of homogeneous leukoplakia, and that the risk of malignant development was higher (OR = 5.4) for lesions larger than 200 mm^2^; however, no other variables such as the presence of any degree of epithelial dysplasia, site, demarcation, smoking, and surgical intervention were statistically significant factors for the development of malignancy.

Brouns et al. [12] attempted to identify in a retrospective study the factors possibly predictive of MT in a well-defined cohort of 144 patients with long-term follow-up. The mean follow-up period was 51.2 months. They found 11% MT, and the annual rate of MT was approximately 2.6%. A large lesion size (≥ 4 cm) proved to be the only statistically significant predictor of MT.

According to Warnakulasuriya et al. [4], the features that stand out as significant determinants contributing to the malignant potential of OL include advanced age, female sex, leukoplakia exceeding 200 mm^2^, non-homogeneous type (e.g., erythroleukoplakia), and higher grades of dysplasia. More recently, Aguirre et al. [5] reported that female sex, non-homogeneous clinical type, and the presence of epithelial dysplasia were significantly related to MT. Other risk factors suggested previously did not show significant results.

However, as we have indicated and as various authors pointed out, although a series of factors predispose to the development of cancer, this does not mean that a homogeneous leukoplakia or a leukoplakia without dysplasia cannot progress to cancer, as pointed out by Villa and Woo [16]. In our study, for example, 7.2% of the homogeneous leukoplakias developed into cancer, while MT occurred in 30.2% of the non-homogeneous forms. We also found that 7.7% of leukoplakias without dysplasia developed into cancer, while MT occurred in 25.9% with mild dysplasia, 36.4% with moderate dysplasia, and 50% of severe forms.

It is evident that MT is much higher and statistically significant in cases that can be considered at higher risk, such as non-homogeneous leukoplakias and high-grade dysplasia; however, we have not found in the literature a study taking into account the mixed nature of OL patients. Therefore, past studies were not able to determine, specifically for the susceptible fraction of the population, the time it takes for these different clinical and histological forms to develop MT. It is important to note that ignoring this mixed nature of the population is a potential cause of bias in estimations of the hazard ratios in Cox regression. The main objective of this article was to use an appropriate model for this specific situation. For this, we adjusted a cure model that considers that the population is a mixture of susceptible and non-susceptible individuals and first estimates the probability of cure (incidence model) and then makes an inference on the time to event in the susceptible individuals (latency model). With this approach, we found that histology type, location in the tongue, and, probably, clinical type are factors that, in susceptible individuals (those who will develop cancer), increase the instantaneous risk of MT, thus reducing the time needed to develop cancer from OL.

We would like to emphasize that it is accepted in Europe that non-homogeneous leukoplakias with no/mild dysplasia are often not treated with surgery but kept under observation. It is also true that some patients ask us for surgical removal of the lesions when, above all, there are histological signs of moderate epithelial dysplasia and in this study we decided to select only those patients treated surgically, but obviously we have other patients who are simply kept under observation.

In conclusion, the time to develop cancer is shorter in patients with non-homogeneous leukoplakia compared to homogeneous OL that transforms into cancer, in those with high grades of epithelial dysplasia compared with those without dysplasia that ended in cancer, and in those with OL on the tongue compared to those with OL not located on the tongue.

The concrete consequences for the clinician are that although non-homogeneous leukoplakias, those with a high degree of dysplasia and localized leukoplakias have a greater tendency to develop cancer, the clinician should never fail to take into account and review patients with homogeneous leukoplakias and those without dysplasia because although less frequently they can also end in cancer, which means that in our view, all leukoplakias should be followed up by the clinician, whether they are treated or not.
